# A novel NPHS1 variant in a Chinese infant with congenital nephrotic syndrome: a case report and literature review

**DOI:** 10.3389/fped.2025.1632898

**Published:** 2025-10-14

**Authors:** Wei Zhang, Li Min Hou, Xian Cheng

**Affiliations:** ^1^ Department of neonatology, Dalian Women and Children's Medical Group, Dalian City, Liaoning Province, China; ^2^ Department of Pediatric Orthopaedic, Dalian Women and Children's Medical Group, Dalian City, Liaoning Province, China

**Keywords:** congenital nephrotic syndrome (CNS), NPHS1, Finnish type, Chinese, Asian

## Abstract

Congenital nephrotic syndrome is a rare autosomal recessive genetic disorder, with the Finnish type caused by NPHS1 variants being the most common. It is characterized by massive proteinuria, hypoalbuminemia, hypercholesterolemia, and edema, ultimately progressing to end-stage renal disease. To date, 260 genetic variants in NPHS1 have been reported, with two variants prevalent in Finnish patients, while non-Finnish populations exhibit greater genetic heterogeneity. This case report describes a Chinese Han female neonate with Finnish-type CNF, highlights the characteristics of her gene variants, and discusses the genetic heterogeneity of Finnish-type CNF between Asian and Finnish populations.

## Introduction

Congenital nephrotic syndrome (CNS) is a rare and severe renal disorder with autosomal recessive inheritance, affecting 1–3 per 100,000 live births globally (1). It is significantly more prevalent in the Finnish population, where Finnish-type Congenital Nephrotic Syndrome (CNF) occurs in approximately 1 in 8,200 live births, with NPHS1 (19q13.1) gene variants accounting for 98% of cases (2–4). Genetic screening for NPHS1 in non-Finnish patients reveals lower variant frequencies but greater heterogeneity. Over 260 NPHS1 variants have been reported, including protein-truncating non-sense variants, frameshift insertions/deletions, and splice site changes. *In vitro* functional studies show that most NPHS1 missense variants cause protein retention in the endoplasmic reticulum, leading to complete loss of cell surface nephrin (5, 6). We present a female neonate with CNF carrying heterozygous NPHS1 variants, including two confirmed pathogenic variants and one variant of uncertain significance.

## Case presentation

A female Han neonate was admitted to the neonatal intensive care unit at 24 days of life with “groaning and poor responsiveness for 18 h.” She was the second pregnancy (G2P1), born at 34 + 6 weeks via cesarean section after 8 h of premature rupture of membranes. The placenta was large (1,300 g), and birth weight was 2,900 g. Urine had been persistently yellow since birth. Prenatal ultrasound revealed bilateral renal enlargement and increased parenchymal echogenicity. Family history: The 33-year-old mother had gestational diabetes and an immune-related disorder, treated with aspirin and low-molecular-weight heparin. She had one prior pregnancy loss due to umbilical cord blood flow interruption. The father was healthy. The parents were non-consanguineous, with no family history of genetic or renal diseases.

### Physical examination

The infant had a term appearance, with poor responsiveness, shallow and irregular breathing with mild intercostal retractions. Other findings include pale complexion, anterior fontanelle 6.0 × 6.0 cm^2^ (slightly bulging), clear lung sounds, regular heart rate at 192 bpm, liver palpable 1 cm below the costal margin (soft texture), non-palpable spleen, cool extremities, capillary refill time of 5 s, and normal primitive reflexes.

### Laboratory findings

Urinalysis: occult blood 2 + cells/μL, protein 3 + g/L, glucose 4 + mmol/L. Blood biochemistry: ALT 4 U/L, prealbumin 80 mg/L, total protein 26.0 g/L, albumin 9.3 g/L, glucose 5.62 mmol/L, cholesterol 7.38 mmol/L, GGT 160.56 U/L. Urine biochemistry: β2-microglobulin 8,196.5 μg/L, creatinine 0.036 g/L. Protein quantification: 10,370.8 mg/L, microalbumin 939.51 mg/dl. Blood tests/inflammatory markers: WBC 5.01 × 10^9^/L, Hb 138 g/L, platelets 331 × 10^9^/L, CRP 132.99 mg/L, PCT 75.89 ng/mL. Cultures: blood and cerebrospinal fluid cultures grew *Streptococcus agalactiae*. Hepatitis markers were negative.

### Imaging

Renal ultrasound showed enlarged kidneys (left 6.8 cm × 2.7 cm, right 6.0 cm × 2.7 cm) with increased parenchymal echogenicity and indistinct corticomedullary junction. Brain MRI revealed abnormal signals in the left occipital cortex and subcortex (local brain injury) and prominent subarachnoid spaces around the bilateral frontotemporal lobes and cerebellum.

### Genetic testing

With parental consent, whole-exome sequencing was performed. The Roche KAPA HyperExome panel was employed to capture and enrich DNA in targeted exonic regions and adjacent splicing junctions, covering approximately 20,000 gene exonic regions in the human genome along with the mitochondrial genome. Variant detection was performed using the MGISEQ-2000 sequencing platform, with sequencing reads aligned to the University of California Santa Cruz (UCSC) hg19 human reference genome via BWA software, followed by duplicate removal. Integrating clinical data from subjects, variant annotation, and filtration were conducted utilizing population databases (including ClinVar, ESP6500, and 1000 Genomes Project), disease-specific databases (such as Online Mendelian Inheritance in Man), and bioinformatics prediction tools (including SIFT and MutationTaster).

**Figure 1:**
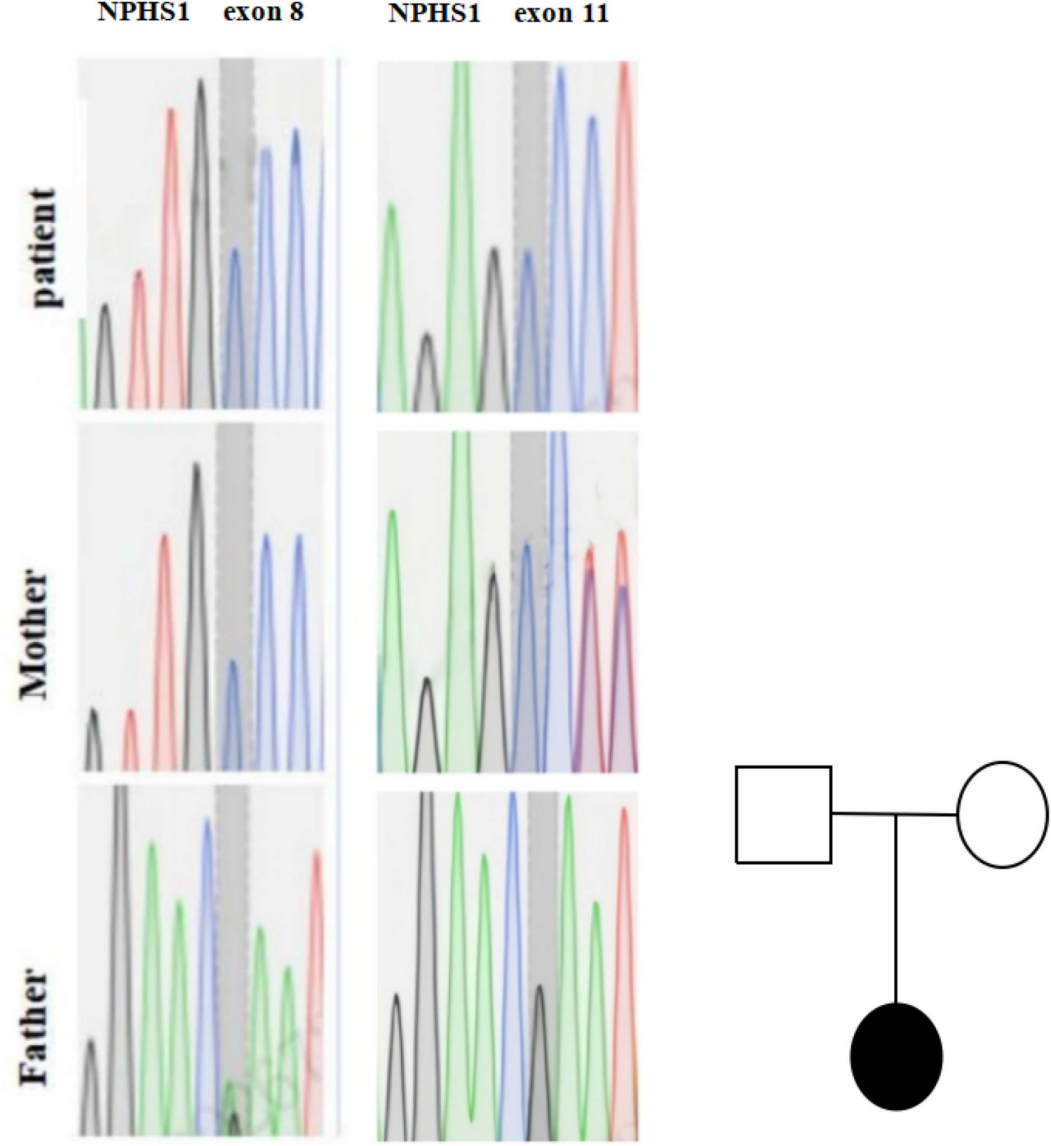
Genetic testing results of the proband's family. Genetic sequencing chromatograms for NPHS1 gene mutations in exon 8 and exon 11 for a patient, mother, and father. The chromatograms show varying peaks representing different nucleotides. On the right is a pedigree chart with a square for the father, a circle for the mother, and a filled circle forthe patient, indicating the patient is affected.

Results identified heterozygous NPHS1 variants inherited from both parents (Table 1 and Figure 1). Per American College of Medical Genetics and Genomics (ACMG) guidelines, c.2515delC (p.Gln839Argfs) in exon 8 of chr19 is a reported pathogenic variant; c.1422delC (p.Ser475Profs) in exon 11 is a novel pathogenic variant. A VUS, c.3337G>A (p.Glu1113Lys) in exon 26, was also detected. Suspected pathogenic variants were identified in NEK8 (c.222_223insA, p.Ala75Serfs) and TNFRSF13B (c.226G>A, p.Gly76Ser; c.41G>A, p.Arg14His), with the latter linked to immune deficiencies in the literature (7, 13).

**Table 1:** Main genetic test results.

Gene name	Variant site	Inheritance pattern	Pathogenicity	Prediction of parental origin
NPHS1	c.1422delC	Paternal origin and heterozygous status	Yes	Father
NPHS1	c.2515delC	Maternal origin and heterozygosity	Yes	Mother
NEK8	c.222_223insA	Maternal origin and heterozygosity	Suspected	Mother
TNFRSF13B	c.226G>A	Biallelic heterozygosity	Uncertain significance	One for each parent
TNFRSF13B	c.41G>A	Biallelic heterozygosity	Uncertain significance	One for each parent

**Diagnoses:** (1) CNF, (2) neonatal purulent meningitis, (3) neonatal septicemia, (4) septic shock, (5) neonatal anemia. With parental consent, whole-exome sequencing was performed.

The infant received comprehensive treatment, including antibiotics, albumin infusions, angiotensin-converting enzyme (ACE) inhibitors, diuretics, and fluid balance management. However, she was discharged after 18 days due to parental decision. She had persistent hypoalbuminemia, edema, and proteinuria, with subsequent hospitalizations for infections and hypocalcemic convulsions until November 2022.

The patient was discharged with persistent hypoalbuminemia, accompanied by edema and proteinuria, and continued to receive conservative management. Occasional infections, including pulmonary edema and enteritis, were observed. Intermittent dialysis was performed, and the patient remained alive with normal renal function.

## Discussion and conclusions

Genetic testing identified a heterozygous variant in exon 8 of chr19 (c.2515delC) from the mother, confirming Finnish-type CNS per ACMG criteria. A novel pathogenic variant (c.1422delC) in exon 11 and a VUS (c.3337G>A) were also found. Suspected pathogenic variants in NEK8 and TNFRSF13B require further validation.

### Genetic testing characteristics in Finnish populations

CNF incidence in Finns is ∼1/8,200 live births, with NPHS1 variants accounting for 98% of cases (6, 8, 9). The “Finmajor” (c.121delCT, p.L41fs) and “Finminor” (c.3325C>T, p.R1109X) variants comprise 78% and 16% of mutant alleles, respectively, vs. 39%–55% in non-Finns. The nephrin protein (1,241 amino acids) is critical for glomerular filtration; its deficiency leads to slit diaphragm dysfunction, mesangial sclerosis, and protein transduction domain (PTD) microcystic dilation, progressing to ESRD (5, 10).

### Genetic testing characteristics in Asian populations

Non-Finnish CNS cases exhibit >260 NPHS1 variants, with genetic heterogeneity contributing to unclear etiologies in 20% of cases (11, 14). Asian patients more commonly have compound heterozygous variants (72%) vs. Finnish homozygous variants (65% Finmajor homozygote). For example, Japanese studies report NPHS1 (4/13 cases) and NPHS2 (2/13 cases) variants, while Chinese cases show diverse heterozygous variants (12, 14–23).

In summary, this report describes a Chinese Han neonate with CNF harboring a novel NPHS1 variant (c.1422delC), expanding the genetic and phenotypic spectrum of CNS. These findings contribute to enhanced molecular diagnosis, genetic counseling, and prenatal testing for CNS.

## Data Availability

The raw data supporting the conclusions of this article will be made available by the authors, without undue reservation.
